# Examining the Effects of Brief Mindfulness Training on Athletes' Flow: The Mediating Role of Resilience

**DOI:** 10.1155/2021/6633658

**Published:** 2021-05-24

**Authors:** Fengbo Liu, Zhongqiu Zhang, Shuqiang Liu, Nan Zhang

**Affiliations:** ^1^School of Psychology, Beijing Sport University, Beijing, China; ^2^China Institute of Sport Science, Beijing, China

## Abstract

**Background:**

Flow is characterized by the strong concentration in competitions, eliminating irrelevant thoughts and emotions, integrating all tasks, and continuing the competition smoothly even in challenging situations. The present study was into whether or not brief mindfulness training can improve athletes' flow and further explore the mediating effect of resilience in the intervention.

**Methods:**

The 2 (experimental conditions) × 2 (time) mixed design was used in this study. Fifty-seven student-athletes were recruited and randomly assigned into either a brief mindfulness group (*n* = 29) or a control group (*n* = 28). Before and after the intervention, every participant completed a self-report measure including mindfulness, flow, and resilience.

**Results:**

Participants in the brief mindfulness group showed increased mindfulness, flow, and resilience (*p* < 0.001) after brief mindfulness training; when putting resilience change (*B* = 0.30, 95% CI [0.031, 0.564]) into the equation, the direct (95% CI [3.156, 13.583]) and indirect (95% CI [0.470, 5.048]) effects of mindfulness training were both significant.

**Conclusion:**

It was concluded that brief mindfulness training could significantly improve athletes' flow and resilience, and resilience partly mediated the effects of brief mindfulness training on flow.

## 1. Introduction

With the continuous improvement of science, technology, and training level, the differences in techniques and tactics of athletes are gradually reduced. Therefore, the psychological state has been regarded as the key factor for athletes to win in competitions and has continuously attracted the attention of researchers. “The optimal competitive state” theory [1] also pointed out that the optimal psychological state was the basis for athletes to compete on the court. With the aim of improving athletes' psychological state (e.g., flow or well-being) and managing their mental health disorders (e.g., anxiety or depression), mindfulness has been integrated into cognitive behavioural training in the sport context [2]. Compared to traditional cognitive behavioural training (e.g., relaxation training and imagery training), which builds on the rationale of controlling or changing the contents of performers' undesirable psychological events in order to achieve the optimal psychological states [3], mindfulness training is an alternative approach for individuals to experience their psychological events (i.e., perceptual experiences). In a state of mindfulness, individuals apply an accepting and nonjudging approach to act and think rather than trying to change or control those experiences [4]. Therefore, mindfulness training encourages individuals to pay attention to the present, which helps disengage distractions from their ruminative states. In other words, mindfulness training may help individuals avoid ineffective or counterproductive psychological states [5].

Athletes' optimal psychological state that has been most discussed in recent years is “flow.” Flow is characterized by strong concentration in competitions, eliminating irrelevant thoughts and emotions, integrating all tasks, and continuing the competition smoothly even in challenging situations [6], which has an important positive effect on the performance of athletes. Therefore, how to increase athletes' flow has become an important topic in the field of sports.

The benefits of mindfulness training on flow have been explained by recent studies [7]. Theoretically, it has been proposed that mindfulness (e.g., brief meditation) can help individuals keep their attention in the present and enhance their body awareness and emotion regulation [8], while the purpose of flow is to develop the consciousness or emotion which is helpful for their optimal performance through devoting themselves to the task [9]. With regard to the empirical evidence on the mechanisms of attention control and emotion, it has been demonstrated that mindfulness training can cause a structural change of grey matter in the brain [8]. As such, mindfulness training might well be useful for increasing flow because of its neurological effects related to psychological effects, such as attention and emotion control [10], which both are the basis of flow.

Although there has been some evidence on the link between mindfulness and athletes' flow [3], the application of brief mindfulness training on flow and its mediating mechanism has not been researched. It was found that when discussing athletes' mindfulness and flow, it was always associated with resilience [11]. Resilience refers to the good adaptation process of human beings in the face of adversity, trauma, or stress, which is an important quality of athletes [12]. The competition in sports is very fierce, so the adversity and stress experience of athletes is quite common. A number of empirical studies have shown that mindfulness training can improve athletes' resilience [13, 14]. The reason is that the concept of mindfulness is acceptance, nonjudgment, and paying attention to the present. Thus, athletes can still focus on the task when facing threats or challenges in their training or competitions and can adapt to changes from the external environment; that is, the level of resilience is improved.

In addition, when the athlete's ability to adapt to threats and challenges is improved, they can get rid of the fear of failure, form a positive evaluation of their own performance, and experience more positive emotions such as self-realization and psychological satisfaction [15]. According to the extended construction theory of positive emotions, the above-mentioned positive emotion experience can enhance athletes' cognitive flexibility and make them more creative in methods exploration of emotion regulation, which are all helpful for enhancing pathway thinking and improving resilience [16]. As flow is characterized by eliminating irrelevant thoughts and emotions, therefore, the increase of flow is more predictable. That is, by mindfully accepting experiences instead of perseverating on them, cognitive resources are freed up to broaden the scope of attention to encompass pleasurable and meaningful events [17] and thereby build motivation toward purposeful engagement with competitions, which is helpful for increasing athletes' flow.

The majority of previous studies had focused on the effects of long-term mindfulness training on flow and resilience [13, 18], and a few intervention studies had explored the relationship between brief mindfulness, flow, and resilience for athletes; thus, the adoption of brief mindfulness training in this study is purposeful and fills a missing research gap. Clearly, there could be potential confounding factors arising from prolonged mindfulness interventions, such as improved attentional control [19], which makes it difficult for singling out mindfulness practice as the cause of improved flow [20]. Here, evidence of whether a brief mindfulness training affects the flow of athletes reveals the effects more directly. Furthermore, some scholars highlighted the potential difficulty in getting athletes to use mindfulness strategies effectively [21]. Long-term mindfulness training, for example, has been known to be easy for participants to drop out, particularly for athletes [22]. Currently, research evidence suggests the effectiveness of brief mindfulness training in eliciting positive outcomes, such as goal motivation and tolerance to negative affect [14]. The effects of such brief interventions led us to consider whether a brief mindfulness training would similarly improve athletes' flow and resilience.

Previous studies have examined the changes in flow and resilience before and after mindfulness training [13, 18]. However, the mediating mechanism by which mindfulness training improves athletes' flow has rarely been explored. In addition, our efforts in testing the effects of brief mindfulness training over a short duration of 30 minutes hopefully contribute to the development of a simple strategy that can be readily applied by athletes without expectation for prolonged sitting meditation. Therefore, the aim of the present study was to examine the effects of brief mindfulness training on athletes' flow and its mediating mechanism. Based on the presented theoretical review we developed the following hypotheses: (1) brief mindfulness training can improve the level of flow among athletes; (2) brief mindfulness training can improve the level of resilience among athletes; (3) resilience plays a mediating role between brief mindfulness training and flow; that is, brief mindfulness training can increase athletes' flow through improving their resilience.

## 2. Methods

### 2.1. Participants

Ethics clearance was granted by the research ethics committee of the Beijing Sport University. Bühlmayer reported a medium-to-large effect size of the mindfulness effect for athletes [23]. An a priori power analysis determined that we would need a total sample size of 52 participants to detect this effect size (G∗power; effect size *f* = 0.35, *α* = 0.05, 1−*β* = 0.80, Corr among rep measures = 0.50).

All participants were recruited from a sport university in China, who were all above level 2 and right-handed, had a normal or corrected-to-normal vision, and did not have any mindfulness training experience. In total, 60 student-athletes (20 females and 40 males) participated voluntarily in this study. Participants were randomly separated into two groups: the brief mindfulness group (*n* = 30) and the control group (*n* = 30) using a random number generator.

Due to time commitment, 29 of 30 athletes in the brief mindfulness group (9 females and 20 males; age average = 19.9, *SD* = .7) and 28 of 30 athletes in the control group (9 females and 19 males; age average = 19.5, *SD* = .8) completed the study. Written informed consent was provided to participants before inclusion and the confidentiality and anonymity of their participation were assured.

### 2.2. Procedure

A 2 × 2 mixed factorial design was employed, with the group (brief mindfulness group vs. control group) as a between-subject factor, and the time (preintervention vs. postintervention) as a within-subject factor. All participants were tested individually. Before the experiment, each participant completed a brief demographic questionnaire to assess age, sex, and meditation experience. Afterward, each participant completed a self-report measure of mindfulness, flow, and resilience and was then randomly assigned to one of the two groups.

Participants in the brief mindfulness group were seated in an empty classroom and were instructed to listen to a mindfulness training audio recording (30 minutes) and complete the exercises outlined in the audio recording. The brief mindfulness training recording was recorded in advance by a mindfulness instructor who has more than 6 years of mindfulness intervention experience. All participants were intervened based on the Gardner and Moore MAC Protocol [2]. The instructor introduced the MAC approach and provided an explanation of the fundamental concepts of mindfulness. The instructor then led the brief centering exercise, where participants attended to their breath and switched their attention to their surroundings, then back to their body. Participants then were asked to complete the body scan exercise, in which participants attended to their breath then progressively moved their attention from one area of the body to another. The goal is to learn how to flexibly move their attention between internal and external sensations and redirect their attention from internal processes (emotions or thoughts) to an external task [14].

Participants in the control group were instructed to listen to a neutral news audio recording for the same duration (30 min). Following the mindfulness or neutral recording, every participant completed a self-report measure of mindfulness, flow, and resilience.

Finally, the fidelity of the intervention was emphasized through the adherence to the protocol, providing uniform delivery to all the participants, observing participant responsiveness and engagement in the intervention, and careful collaboration between the researchers, athletes, and coaches with the goal of determining the elements of the intervention that were essential for its success [24].

### 2.3. Measures

Mindfulness was measured using the Chinese version [25] of the Five Facet Mindfulness Questionnaire (FFMQ) [26], assessing five facets of mindfulness: observing, describing, acting with awareness, nonjudging of inner experience, and nonreactivity to inner experience. Participants rated the 39 items (e.g., “I notice the smells and aromas of things”) of FFMQ by using a five-point Likert scale (1 = never or very rarely true; 5 = very often or always true). For the total measure, scores can range from 39 to 195, with higher scores representing higher levels of mindfulness. The internal consistency reliability of FFMQ was satisfactory across our assessments (*α* = 0.67 to 0.81).

Flow was measured using the Chinese version [27] of Dispositional Flow Scale 2 (DFS-2) [28], assessing nine facets of flow: challenge skill balance, action awareness, clear goals, unambiguous feedback, concentration on task, sense of control, loss self-consciousness, transformation of time, and autotelic experience. Participants rated the 33 items (e.g., “I feel I am competent enough to meet the high demands of the situation”) of DFS-2 by using a five-point Likert scale (1 = never; 5 = always). Their responses were summed to create flow scores. Scores can range from 33 to 165, with higher scores indicating a greater frequency of flow. The internal consistency reliability of DFS was satisfactory across our assessments (*α* = 0.74 to 0.87).

Resilience was measured using the Chinese version [29] of the Resilience Scale (RISC) [30], assessing three facets of resilience: toughness, strength, and optimism. Participants rated the 25 items (e.g., “I can adapt to changes”) of RISC by using a 5-point Likert scale (0 = never; 4 = always). Their responses were summed to create resilience scores. Scores can range from 0 to 100, with higher scores indicating greater levels of resilience. The internal consistency reliability of RISC was satisfactory across our assessments (*α* = 0.63 to 0.87).

### 2.4. Data Analysis

Independent-sample *t*-tests were used to compare the preintervention scores of the brief mindfulness group and control group. Two-way repeated-measures analyses of variance (ANOVA) were conducted to test the effect of time (within-subject independent variable: levels = preintervention and postintervention) and group (between-subject independent variable: levels = brief mindfulness group and control group) on mindfulness, flow, and resilience, respectively. Post hoc simple effect analysis was used to investigate whether there was any difference between pretest and posttest of each group. The macro program PROCESS of SPSS compiled by Hayes [31] was used to test the mediating effect of resilience between brief mindfulness training and flow. The number of Bootstrap samples was 5000. Under the 95% confidence interval, postintervention score of flow was used as the dependent variable, the group was used as the independent variable, and the score change of resilience (postintervention minus preintervention) was used as the mediating variable. Since the postintervention score of flow was affected by preintervention score, therefore, gender, age, and preintervention score of flow were all controlled as covariates.

## 3. Results

In preliminary data screening, no missing data were observed. See Table 1 for the means, standard deviations, and preintervention comparison of the study variables of each group.

**Figure 1:**
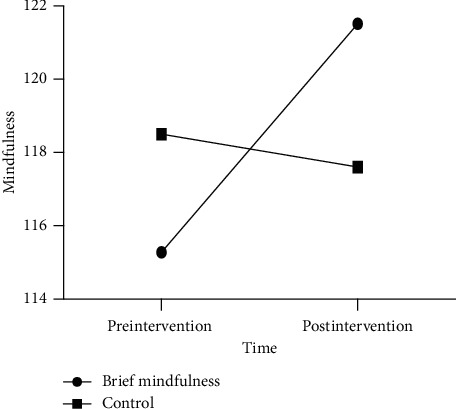
Mindfulness scores of two groups in pre- and postintervention.

Repeated-measures ANOVA of mindfulness revealed a significant time by group interaction effect, *F* (1, 55) = 13.75, *p* < 0.001, *η*_*p*_^*2*^ = 0.200, and within-subject difference of mindfulness across time was significant, *F* (1, 55) = 7.73, *p*=0.007, *η*_*p*_^*2*^ = 0.123. Furthermore, a between-group difference of mindfulness was not significant, *F* (1, 55) = 0.02, *p*=0.886, *η*_*p*_^*2*^ < 0.001; see Table 2. Further simple effect analysis indicated that the mindfulness level of the brief mindfulness group was significantly higher in postintervention, as compared to that of preintervention (*p* < 0.001), while the mindfulness level of the control group was a little lower in postintervention, as compared to that of preintervention (*p*=0.518); see Figure 1.

**Figure 2:**
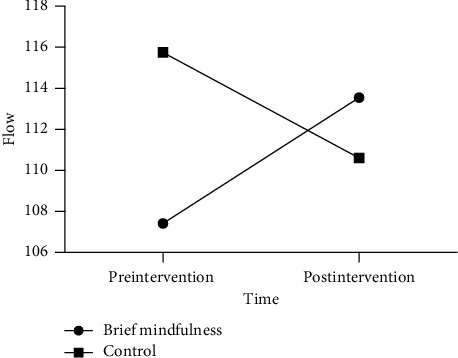
Flow scores of two groups in pre- and postintervention.

Repeated-measures ANOVA of flow revealed a significant time by group interaction effect, *F* (1, 55) = 23.17, *p* < 0.001, *η*_*p*_^*2*^ = 0.296, and within-subject difference of flow across time was not significant, *F* (1, 55) = .18, *p*=0.673, *η*_*p*_^*2*^ = 0.003. Furthermore, a between-group difference of flow was not significant, *F* (1, 55) = 0.68, *p*=0.413, *η*_*p*_^*2*^ = 0.012; see Table 2. Further simple effect analysis indicated that the flow of brief mindfulness group was significantly higher in postintervention, as compared to that of preintervention (*p* < 0.001) while the flow of control group was significantly lower in postintervention, as compared to that of preintervention (*p*=0.003). Hypothesis 1 was verified; see Figure 2.

**Figure 3:**
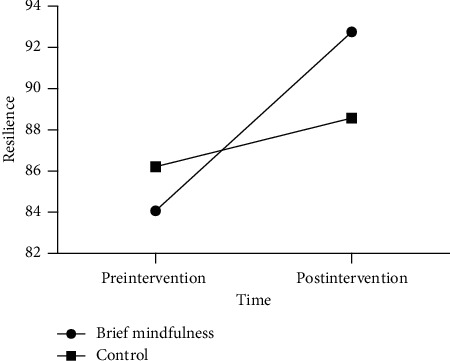
Resilience scores of two groups in pre- and postintervention.

Repeated-measures ANOVA of resilience revealed a significant time by group interaction effect, *F* (1, 55) = 7.91, *p*=0.007, *η*_*p*_^*2*^ = 0.126, and within-subject difference of resilience across time was significant, *F* (1, 55) = 24.08, *p* < 0.001, *η*_*p*_^*2*^ = 0.306. Furthermore, a between-group difference of resilience was not significant, *F* (1, 55) = 0.11, *p*=0.743, *η*_*p*_^*2*^ = 0.002; see Table 2. Further simple effect analysis indicated that the resilience of the brief mindfulness group was significantly higher in postintervention, as compared to that of preintervention (*p* < 0.001) while the resilience of the control group was a little higher in postintervention, as compared to that of preintervention (*p*=0.148). Hypothesis 2 was verified; see Figure 3.

From Table 3, it can be found that the total effect of the group on postintervention flow was significant (*B* = 10.38, 95% CI [5.302, 15.457]); resilience change had a significant predictive effect on postintervention flow (*B* = .30, 95% CI [0.031, 0.564]). Further analysis found that after the mediating variable entered the equation, the indirect effect Bootstrap 95% CI [0.470, 5.048] did not contain 0; besides, the direct effect bootstrap 95 % CI [3.156, 13.583] did not contain 0. Hypothesis 3 was verified.

## 4. Discussion

The current study provides preliminary evidence for the effectiveness of brief mindfulness training on mindfulness, flow, and resilience for athletes. Compared with the control group, athletes who completed the brief mindfulness training significantly improved their mindfulness, flow, and resilience at the postintervention test, indicating the positive effects of brief mindfulness training for athletes.

First, we predicted that brief mindfulness training would increase levels of trait mindfulness, as measured by total FFMQ scores, compared with the control group. On the one hand, total FFMQ scores were greater in the brief mindfulness group compared with the control group, which is consistent with our hypothesis and provides additional support for previous studies that used brief mindfulness training [32]. On the other hand, considering that baseline total FFMQ scores did not significantly differ across groups, it is unlikely that these results are simply due to preintervention differences in trait mindfulness across groups. This finding is consistent with previous research [32] and demonstrates the mindfulness benefits associated with brief mindfulness training programs.

Mindfulness has been linked to significant and positive relations with flow in previous correlational and intervention studies [18, 33]. As shown in our study, the brief mindfulness group had consistently reported higher flow than the control group, which is consistent with our hypothesis. The results validated the positive impact of the brief mindfulness intervention on flow, again. Previous studies showed that the process of mindfulness training emphasizes individuals' perception of inner experience, including emotion, thought, and intention [34], that is, focusing on actions that need to be completed and living in the present, which was the basis of flow.

Our findings are also broadly consistent with the results of the previous research [35]. These latter authors found that athletes who scored highly in mindfulness reported greater scores for some flow dimensions (e.g., sense of control, action awareness, concentration on task) than did athletes with lower mindfulness scores. Our results are understandable given that these dimensions of flow are related to the self-regulation of attention [36]. Thus, with heightened self-regulation of attention, those who are mindful are more likely to concentrate on their tasks and be aware of their action. Likewise, according to Bishop et al. [36] contention, the improvement of the sense of control is also related to self-regulation of attention. However, a noticeable difference is that this study did not find that brief mindfulness training significantly led to increased scores on the flow subscales of “clear goals,” “unambiguous feedback,” and “loss of self-consciousness.” One possible explanation is that the participants were only asked to do a brief centering exercise and body scan exercise in one single-session mindfulness intervention, which was not aiming at improving these three abilities. Further research choosing longer-term interventions and thus greater statistical power will be needed to confirm these findings [18]. It would be premature to conclude that mindfulness does not influence the aspects of flow for which statistically significant effects were not detected in this study.

Moreover, the brief mindfulness group had consistently reported higher resilience than the control group, which is consistent with our hypothesis. The current results showed that brief mindfulness training could help improve athletes' resilience, which meant that brief mindfulness training could improve athletes' receptive ability and avoid being affected by negative events. Previous studies pointed out that the idea of mindfulness originated from Eastern religions and philosophy. It is a concern for nonjudgment of current experience [37], and a “frank awareness”, emphasizing nonjudgment and nonevaluation of the actual situation at present. Although athletes are able to pay attention to the actual situation at present, they will not judge right or wrong, whose purposes are not to control or change their own internal state [38]. Another possible explanation is that mindfulness training facilitates active emotion-focused coping (e.g., cognitive reappraisal). Consistent with this active emotion-focused coping account, one previous study indicates that mindfulness training increases resilience for stressful events [39].

Although positive relations between mindfulness and resilience were shown in previous correlational and intervention studies [40, 41], this study did not find that brief mindfulness training significantly led to increased scores on the resilience subscales of “optimism.” One testable hypothesis is that optimistic coping efforts may be particularly deliberate and effortful after brief mindfulness meditation training but then become more automatic after longer periods of training [42].

Finally, we revealed the mediating mechanism of a brief mindfulness intervention on flow among athletes, which is consistent with our hypothesis. We found that resilience played a partly mediating role in the process of a brief mindfulness intervention improving flow; that is, a brief mindfulness intervention can not only directly improve flow among athletes but also indirectly improve flow through resilience. This reveals the relationship between mindfulness and flow from the perspective of nonjudgment and nonreaction, which is more consistent with the reality of athletes and the “attention control” theory proposed by Diamond [43]. On the one hand, mindfulness emphasizes the psychological characteristics of not judging the past and not worrying about the future. If athletes can be nonjudgmental and nonreactive, they will pay more attention to current tasks and alleviate negative events, which mean the improvement of resilience and are the basis of flow, or they will be immersed in negative events and experiences. On the other hand, mindfulness is a complex psychological state related to the ability of attention control, which can reduce individuals' response to the surrounding environment, and it also means the improvement of resilience and is the basis of flow. This explains why resilience mediates the influence of the brief mindfulness intervention on flow.

While the results of this study are encouraging, several limitations of the present study must be considered. First, we did not examine the follow-up effect of the brief mindfulness intervention. While anecdotal statements from student-athletes a few weeks past the intervention indicated retention of the mindfulness skills, this study did not conduct a formal assessment of the intervention retention. In addition, the sample consisted entirely of student-athletes; thus, whether the conclusion of this study is applicable to elite athletes needs further verification. Future research should include a follow-up with a larger sample size of elite athletes. Moreover, a brief single-session mindfulness intervention was used; thus, this study did not find that mindfulness training led to increased scores on some subscales of mindfulness, resilience, and flow. A longer period of mindfulness training should be used in the future to establish statistically significant changes in every subscale of mindfulness, resilience, and flow when assessed quantitatively. Another limitation is the exclusive reliance on self-report measures. Although the instruments used here have good psychometric support, self-report measures can be subject to biases. Future research may consider including neuron electrophysiology in outcome measures as supplements of an objective indicator of mental status, for example, EEG and MRI.

In summary, brief single-session mindfulness interventions are increasingly being used to examine the effects of mindfulness in a controlled laboratory setting. However, limited brief mindfulness studies have been conducted on athletes' flow. We investigated the degree to which one of these interventions affected measures of flow, and whether the efficacy of the intervention is mediated by resilience. This study expands the functions of brief mindfulness interventions in the field of sports, enriches the mediating mechanism of a brief mindfulness intervention, and focuses on the effects of a brief mindfulness intervention on flow and resilience, which are closely related to athletes' performance and mental health.

## 5. Conclusion

The present study provided initial evidence supporting the application of brief mindfulness training in improving mindfulness, flow, and resilience for athletes. Brief mindfulness training not only appeared to be adaptive to athletes' mindfulness but also seemed to improve their flow and resilience. More importantly, resilience played a mediating role in the process of mindfulness intervention improving athletes' flow. The importance of preserving the habit of regular mindfulness practice after the completion of a brief mindfulness intervention is highlighted.

## Figures and Tables

**Table 1:** Descriptive statistics (M ± SD) of the two groups across preintervention and postintervention and summaries of preintervention independent-sample *t*-test comparison.

Variables	Brief mindfulness group (*n* = 29)		Control group (*n* = 28)		Comparison of preintervention	
	Preintervention	Postintervention	Preintervention	Postintervention	*t*	*p*
Observing	24.83 ± 5.35	27.55 ± 4.85	28.29 ± 5.00	27.75 ± 5.16	−2.51	0.015
Describing	24.07 ± 4.41	25.62 ± 3.82	24.86 ± 4.39	24.11 ± 3.44	−0.68	0.502
Acting with awareness	26.69 ± 4.49	25.17 ± 4.38	24.86 ± 5.67	23.61 ± 5.47	1.35	0.183
Nonjudging	20.03 ± 5.24	19.28 ± 4.23	19.64 ± 4.74	20.07 ± 4.68	0.30	0.768
Nonreactivity	19.66 ± 3.58	23.90 ± 4.50	20.86 ± 3.36	22.07 ± 4.39	−1.32	0.192

*Mindfulness*	115.28 ± 10.97	121.52 ± 9.87	118.50 ± 9.43	117.61 ± 8.37	−1.19	0.239
Challenge skill balance	12.97 ± 2.53	14.10 ± 2.45	14.64 ± 2.78	14.21 ± 3.15	−2.38	0.021
Action awareness	9.52 ± 2.35	10.31 ± 2.04	10.07 ± 2.29	9.57 ± 2.03	−0.90	0.372
Clear goals	11.59 ± 2.26	11.28 ± 2.40	11.57 ± 2.12	11.04 ± 2.15	0.03	0.980
Unambiguous feedback	15.03 ± 2.47	15.07 ± 2.05	15.11 ± 2.20	14.29 ± 2.34	−0.12	0.907
Concentration on task	12.62 ± 2.43	14.03 ± 2.64	13.64 ± 2.21	13.32 ± 2.58	−1.66	0.102
Sense of control	8.90 ± 1.99	9.59 ± 1.35	10.14 ± 1.60	9.61 ± 1.40	−2.61	0.012
Loss self-consciousness	10.62 ± 3.82	11.31 ± 3.80	11.25 ± 2.88	10.57 ± 3.28	−0.70	0.484
Transformation of time	12.76 ± 3.59	13.62 ± 3.59	14.11 ± 3.38	13.50 ± 2.95	−1.46	0.150
Autotelic experience	13.41 ± 2.83	14.24 ± 2.50	15.21 ± 3.00	14.50 ± 3.35	−2.33	0.024

*Flow*	107.41 ± 11.98	113.55 ± 12.76	115.75 ± 12.86	110.60 ± 14.74	−2.53	0.014
Toughness	41.79 ± 7.01	47.31 ± 7.36	44.04 ± 6.60	45.57 ± 7.03	−1.25	0.219
Strength	28.90 ± 3.68	31.14 ± 4.05	28.57 ± 4.35	29.21 ± 5.41	0.30	0.762
Optimism	13.38 ± 2.58	14.31 ± 2.44	13.61 ± 2.31	13.79 ± 2.50	−0.35	0.727

*Resilience*	84.07 ± 11.75	92.76 ± 12.45	86.21 ± 11.95	88.57 ± 13.51	−0.68	0.497

**Table 2:** Summaries of two-way repeated-measures ANOVA comparison.

Variables	Within-subject						Between-subject		
	Time			Time × group			*F*	*p*	*η*_*p*_^*2*^
	*F*	*p*	*η*_*p*_^*2*^	*F*	*p*	*η*_*p*_^*2*^			
Observing	7.65	0.008	0.122	16.966	<0.001	0.236	2.01	0.162	0.035
Describing	0.74	0.395	0.013	6.06	0.017	0.099	0.14	0.708	0.003
Acting with awareness	6.92	0.011	0.112	0.07	0.800	0.001	1.93	0.171	0.034
Nonjudging	0.16	0.691	0.003	2.06	0.157	0.036	0.03	0.865	0.001
Nonreactivity	3.80	<0.001	0.420	12.25	0.001	0.182	0.11	0.747	0.002

*Mindfulness*	7.73	0.007	0.123	13.75	<0.001	0.200	0.02	0.886	<0.001
Challenge skill balance	2.43	0.125	0.042	11.85	0.001	0.177	1.68	0.200	0.030
Action awareness	0.36	0.550	0.007	7.05	0.010	0.114	0.03	0.861	0.001
Clear goals	3.28	0.076	0.056	0.23	0.632	0.004	0.06	0.816	0.001
Unambiguous feedback	2.00	0.163	0.035	2.36	0.130	0.041	0.44	0.508	0.008
Concentration on task	3.04	0.087	0.052	7.68	0.008	0.123	0.07	0.789	0.001
Sense of control	0.13	0.718	0.002	8.33	0.006	0.132	2.95	0.091	0.051
Loss self-consciousness	<0.001	0.989	<0.001	3.13	0.082	0.054	0.01	0.948	<0.001
Transformation of time	0.27	0.605	0.005	8.99	0.004	0.140	0.51	0.477	0.009
Autotelic experience	0.04	0.848	0.001	6.90	0.011	0.112	2.05	0.158	0.036

*Flow*	0.18	0.673	0.003	23.17	<0.001	0.296	0.68	0.413	0.012
Toughness	25.08	<0.001	0.313	7.99	0.007	0.127	0.02	0.884	<0.001
Strength	13.47	0.001	0.197	4.14	0.047	0.070	1.04	0.311	0.019
Optimism	5.30	0.025	0.088	2.44	0.124	0.042	0.06	0.807	0.001

*Resilience*	24.08	<0.001	0.305	7.91	0.007	0.126	0.11	0.743	0.002

*Note. η*_*p*_^*2*^: partial *η*^*2*^.

**Table 3:** Summaries of PROCESS mediating effect test.

Dependent variable: postintervention flow	Coefficient/effect	Boot se	95% CI LL	95% CI UL
Independent variables:				
Gender	−4.29	2.42	−9.148	0.568
Age	−0.70	1.56	−3.826	2.428
Preintervention flow	0.85	0.09	0.664	1.029
Δ resilience	0.30	0.13	0.031	0.564
Direct effect	8.37	2.60	3.156	13.583
Indirect effect	2.01	1.09	0.470	5.048
Total effect	10.38	2.53	5.302	15.457

*Note.* CI = confidence interval; LL = lower limit; UL = upper limit; group 1 = control group, group 2 = brief mindfulness group; Δ = postintervention score minus preintervention score.

## Data Availability

The data used to support the findings of this study are available from the corresponding author upon request.
